# The role of titanium surface micromorphology in MG-63 cell motility during osteogenesis

**DOI:** 10.1038/s41598-022-13854-2

**Published:** 2022-06-15

**Authors:** Fang Jia, Shuxiu Wang, Shulan Xu, Wangxi Wu, Lei Zhou, Jingsong Zeng

**Affiliations:** 1 Stomatological Hospital, Southern Medical University, Guangzhou, 510280 China; 2 State Key Laboratory of Pulp and Paper Engineering, Plant Fiber Research Center, South China University of Technology, Guangzhou, 510640 China; 3 Guangdong Plant Fiber High-Valued Cleaning Utilization Engineering Technology Research Center, South China University of Technology, Guangzhou, 510640 China

**Keywords:** Biochemistry, Biological techniques, Biotechnology

## Abstract

Different surface micromorphologies influence osteoblast movements and impact the osteogenesis around implants. In this study, a biomimetic chip that simulates the microenvironment of the implant and bone in vitro was developed (tissue-on-chip of group T and group C) to study the correlation of cell movement velocity (CMV), direction (CMD), acceleration (CMA), and cell attachment number (CA) with the surface micromorphology of the Titanium material. Computational fluid dynamics (CFD) was used for flow analysis. Changes in intraosseous pressure (IOP), local blood perfusion index (LBPI), new bone microstructure, microvessel density (MVD), and bone-implant contact (BIC) in beagle dogs were detected as implant surface alterations. Surface skewness (*Ssk*) and surface arithmetic mean height (*Sa*) were the most important negative factors for high CMV, accounting for 51% and 32%, respectively, of all the influencing factors. Higher *Ssk* (*Ssk*_*T*_ > 0, *Ssk*_*C*_ < 0) and *Sa* (*Sa*_*T*_ > *Sa*_*C*_) resulted in lower CMV (CMV_T_:CMV_*C*_ = 0.41:1), greater CA (CA_T_:CA_C_ = 1.44:1), and higher BIC (BIC_T_:BIC_C_ = 3.06:1) (*P* < 0.05). The surface micromorphology influenced the CMD of MG-63 cells within 20 μm from the material surface. However, it could not regulate the IOP, LBPI, MVD, new bone microstructure, or CMD (*P* > 0.05).

## Introduction

Good osseointegration of tooth implants and bones is vital for the loading function of implants and influences their long-term stability^1,2^. Different implant surface morphologies influence bone-implant contact and osteogenesis^3–8^. Therefore, it is of great significance to study the interaction of osteoblasts and the surface micro-morphologies of different materials in relation to osteoblast movement and attachment.

To this end, microfluidic technology has been used due to the shortcomings of traditional histopathological methods, which can only be used to study the number of cells and cell adsorption at rest^9^. The main research directions of microfluidic chips are microfluidic flow cytometry, separation of circulating tumor cells (CTC), senescent cells, platelets, nucleic acids, exosomes, and sperm^10–18^, as well as the design of 3D cell cultures to observe vascular regeneration and tumor cell colonization^19,20^. These studies suggest the feasibility of 3D bone block applications. A biomimetic model used to simulate the microenvironment of dental implants and bones has not yet been developed.

The use of an improved implant surface treatment scheme (4.62 mol L^−1^ H_3_PO_4_ + 0.249 mol L^−1^ NaF, group T) and the conventional implant treatment scheme (5.00 mol L^−1^ H_2_SO_4_ + 5.05 mol L^−1^ HCl, group C) give rise to two different surface micromorphologies of titanium^21,22^. Therefore, tissue-on-a-chip (TOC: a type of microfluidic chip) consisting of different types of treated titanium sheets has been used to mimic the implant microenvironment in vitro (TOC-T and TOC-C)^23,24^. This study investigated whether changes in the material surface micromorphology affect intraosseous pressure (IOP), local blood perfusion index (LBPI), new bone microstructure, microvessel density (MVD), bone-implant contact (BIC), and the microstructure of new bone around the implant, as well as cell movement velocity (CMV), direction (CMD), and acceleration (CMA). To this end, we combined in vitro and in vivo experiments. This study revealed how the implant surface affects MG-63 cell movement before surface attachment in real-time and whether cell movement influences CA and BIC, thereby elucidating how the implant surface micromorphology affects osteogenesis, which has not been previously reported.

## Results

### Surface skewness (*Ssk*) and surface arithmetic mean height (*Sa*) up-regulate bone-implant contact (BIC)

**Figure 1:**
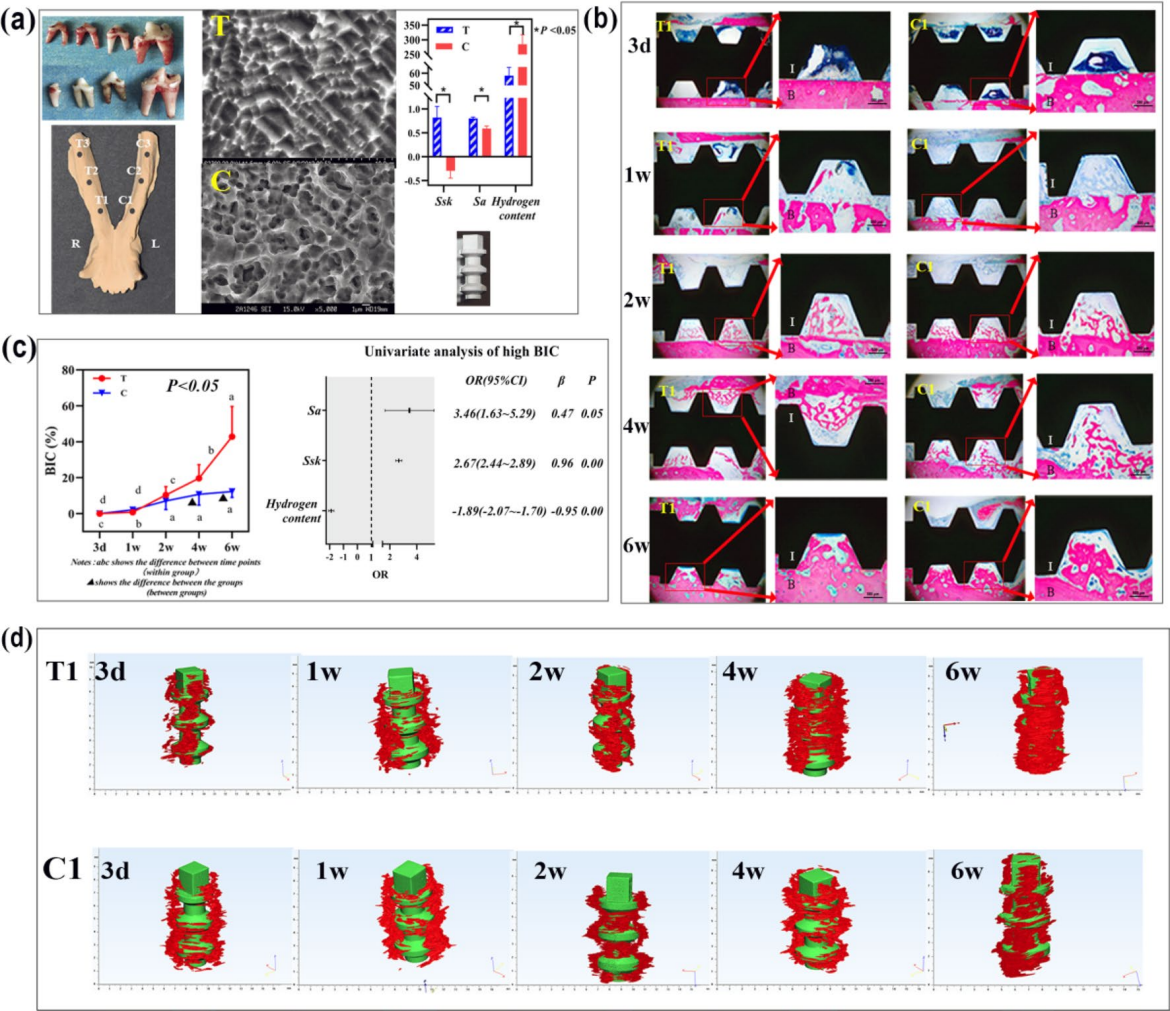
Variations in implant surface micromorphology impact bone-implant contact (BIC). (**a**) Four mandibular premolars of beagle dogs were extracted; SEM images of the surface micromorphology of the implants, etched by either formula T or formula C; the implantation sites. *Ssk*, *Sa* and hydrogen content of implant T and C. (**b**, **c**) Pathological features of new bone adjoined to the implants of groups T1 and C1. The BIC of group T1 was higher than group C1 at 4 w and 6 w after implantation; I: implant; B: bone tissue (*P* < 0.05). *Ssk* and *Sa* resulted in high BIC, accounting for 96% and 47%, respectively, of all factors (*OR* > 1, *β* > 0); the low hydrogen content led to high BIC (*OR* < 1, *β* < 0). (**d**) The quantity of the new bone formed surrounding the implants of group T1 was more than that of group C1 at 4 w and 6 w after implantation (*P* < 0.05). This figure was generated by Adobe photoshop CC 2015.

As shown in Fig. 1a, beagle dogs received two types of dental implants treated with two acid etching formulas (group T and group C, respectively) after the four mandibular premolars were extracted two months previously. The BIC of group T was higher than that of group C at 4 w and 6 w after the implantation (*P* < 0.05) (Fig. 1b,c).

The *Ssk* values of groups T and C were 0.817 ± 0.232 and − 0.297 ± 0.153, respectively. The *Sa* values of groups T and C were 0.80 ± 0.03 $\upmu{\mathrm{m}}$ and 0.59 ± 0.05 $\upmu {\mathrm{m}}$, respectively. The hydrogen contents of groups T and C were 58 ± 6.70 ppm and 285 ± 32.1 ppm, respectively. As shown in Fig. 1c, high *Ssk* and *Sa* values gave rise to high BIC (*OR* > 1, *β* > 0, *P* < 0.05), and *Ssk* (*β* = 0.96, *P* = 0.00) played a more important role than *Sa* (*β* = 0.47, *P* = 0.05). However, the high hydrogen content was a negative and indirect factor that resulted in low BIC (*OR* < 1, *P* = 0.00). *Sq* values (the root mean square of the distribution of relative heights) of groups T and C were 1.04 ± 0.31 μm and 0.78 ± 0.22 μm, respectively, showed the same trend as the *Sa* values (Suppl. Fig. 4).

Figure 1d shows that the quantity of new bone formed next to the implants was greater in group T than in group C at 4 and 6 w after implantation (*P* < 0.05).

### Changes in surface micromorphology did not affect the IOP, LBPI, MVD, or microstructure parameters of new bone formed around the implants

**Figure 2:**
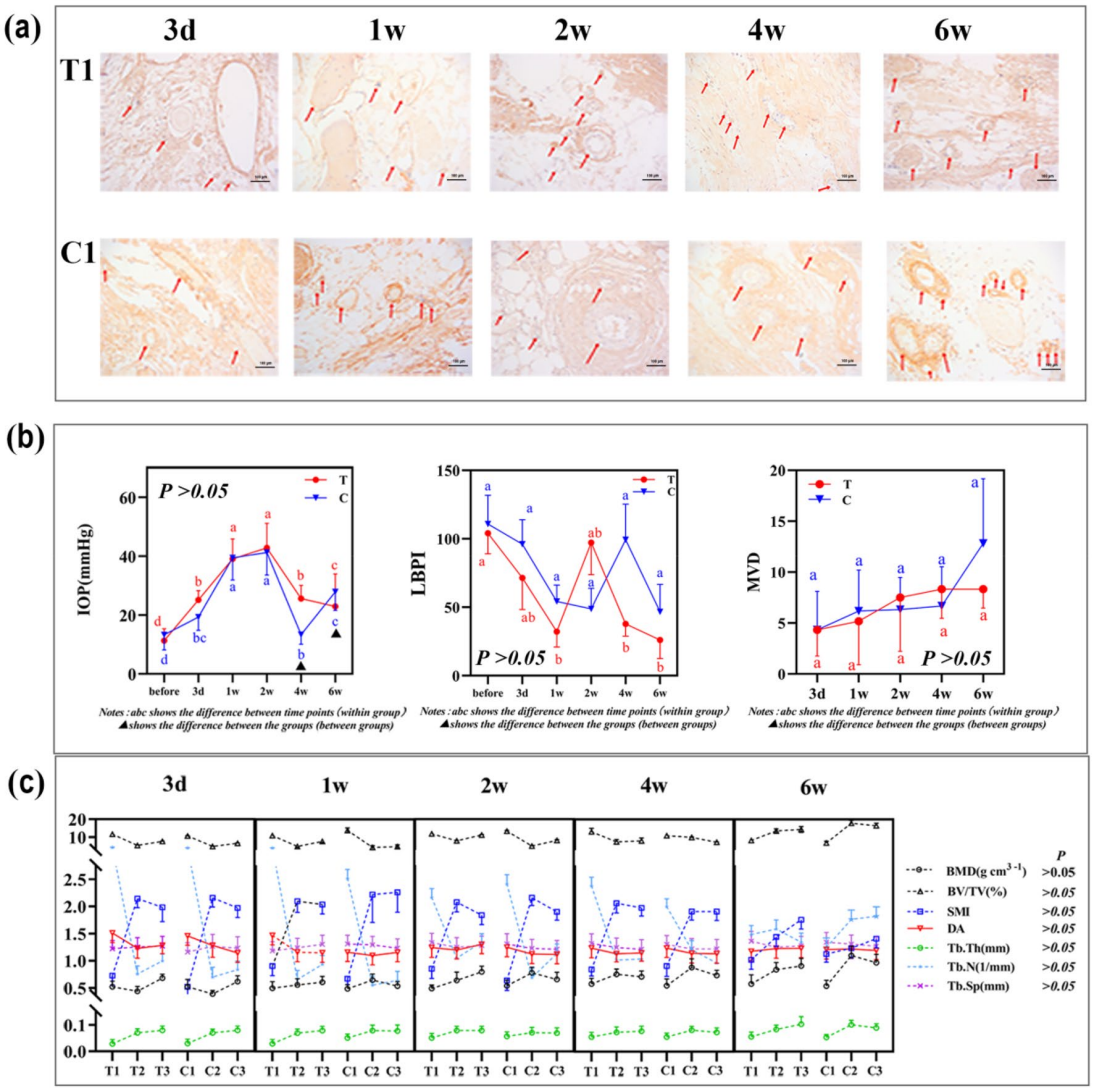
The variation of implant surface morphology had no impact on the intraosseous pressure (IOP), local blood perfusion index (LBPI), or microvessel density (MVD). (**a**) Histopathological images of the newly formed microvessel next to the titanium implants at a magnification of 200${\times}$. The red arrows indicate the newly formed microvessel. (**b**) There was no significant difference in IOP and MVD between groups T and C (*P* > 0.05). (**c**) The major microstructure parameters of the new bone formed surrounding the implants of groups T and C showed no statistical differences. The statistical significance was set at *P* < 0.05. This figure was generated by Adobe photoshop CC 2015.

Supplementary file 3 depicts the test sites and the experimental processes of IOP and LBPI detection. Figure 2a shows the new microvessels formed around the T1 or C1 implants. Statistical analysis showed no significant differences in the IOP, LBPI, and MVD values of groups T and C (*P* > 0.05, Fig. 2b). The IOP diagram showed that there were differences between groups T and C (*P* < 0.05) 4 and 6 w after implantation, but there was no overall significant difference between the two groups (*P* > 0.05). Figure 2c shows that were no significant differences in terms of bone mineral density (BMD), bone volume fraction (BV/TV), structure model index (SMI), degree of anisotropy (DA), trabecular number (Tb.N), trabecular thickness (Tb.Th), and trabecular separating spacing (Tb.Sp) of the new bone formed surrounding the implants between groups T and C (*P* > 0.05). Among those parameters, the BMD values of T1 and C1 were significantly lower than those of T2, T3, C2, and C3 (*P* < 0.05). The SMI values of T1 and C1 were 45% and 41%, respectively, as those of T2, C2, T3, and C3 (*P* > 0.05). The Tb.N values of T1 and C1 were significantly lower, by 55% and 51%, respectively, than T2, T3, C2, and C3 Tb.N values (*P* < 0.05).

### Cell movement assessment

#### Changes in surface micromorphology are affected by CMV, but not CMA

**Figure 3:**
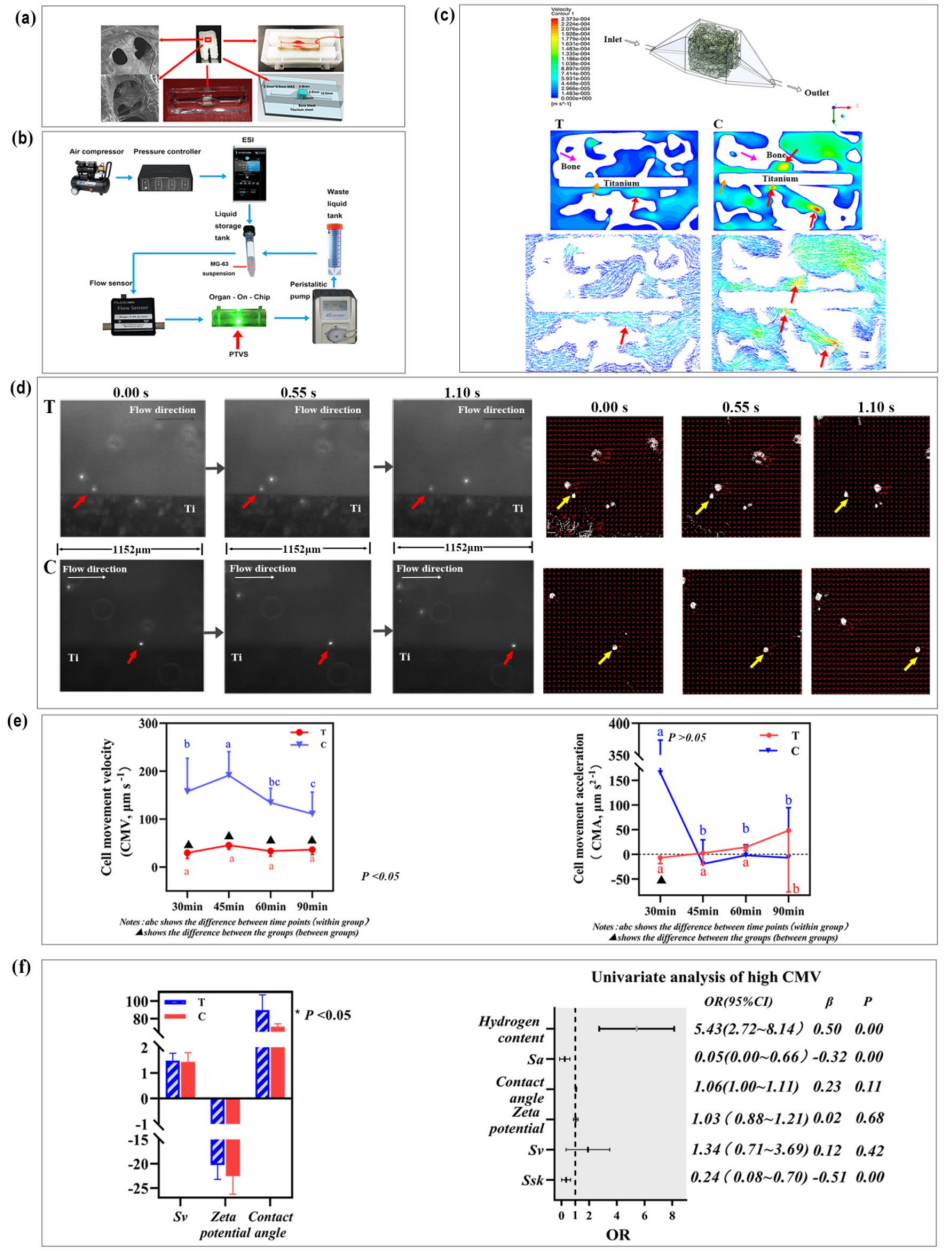
The variation of implant surface morphology affected the cell movement velocity (CMV). (**a**) Design of the tissue-on-chip (TOC). (**b**) The instrument connections of the microfluidic pressure and flow control system. (**c**) 3D reconstruction of the TOC; the velocity contour of the TOC-T and TOC-C, generated using Fluent 19.0. The red arrows indicate that CMV increases when the fluid passes through the smaller cross-sectional area of the bone cavity. (**d**) Live images and velocity vector diagrams of MG-63 cells traversing the TOC-T and TOC-C. (**e**) The CMV of group T was significantly lower than that of group C (*P* < 0.05); there was no significant difference in the CMA of groups T and C (*P* > 0.05). (**f**) The values of *Sv,* contact angle, and zeta potential of groups T and C; Factors contributing to high CMV: *Sa* and *Ssk* negatively influenced the high CMV of MG-63, accounting for − 32% and − 51%, respectively (*P* < 0.05). *Sv*, contact angle, and zeta potential did not affect CMV (*P* > 0.05); the hydrogen content was an indirectly positive factor for high CMV (*P* < 0.05). The statistical significance was set at *P* < 0.05. This figure was generated by Adobe photoshop CC 2015.

Figure 3a shows the tissue-on-a-chip design consisting of acellular canine alveolar bone (ACAB), titanium sheet T or C, and polydimethylsiloxane (PDMS). Scanning electron microscopy (SEM) images showed that the cells in cancellous bone cavities had already been removed. The flow channel and the middle chamber simulated blood diffusion from the inferior alveolar artery into the surrounding bone tissue. As indicated in Fig. 3b, the fluid velocity was controlled by the pressure controller and the flow sensor. The peristaltic pump was used to control the velocity of suspension recirculation.

Figure 3c shows that the overall fluid velocity of group T was much lower than that of group C. When fluid enters the wide chamber from the narrow channel and enters the narrow tunnel from a wide space, the cell movement velocity (CMV) changes. When the cross-sectional area of the chamber in the bone tissue suddenly shrinks, the velocity of the suspension moving far away from the material surface increases, and the CMD changes. However, regardless of the changes in the bone microstructure around the implant, the CMV of MG-63 cells moved close to the material’s surface and the bone wall was unchanged and was the slowest.

As is implied in Fig. 3d and supplementary files 1a and 2a, within 1.10 s, the cell movement distance on the surface of the titanium sheet of group T was 0.41 times that on the surface of the titanium sheet of group C (*P* < 0.05; CMV_C_: 148.74 ± 95.04 μm s^−1^; CMV_T_: 36.14 ± 13.42 μm s^−1^). The motion vector diagram and supplementary files 1b and 2b show that the CMVs of MG-63 on the surface of group T material in all directions were close to each other. In contrast, the movement velocity of cells on the surface of group C material in all directions failed to reach a balance, and the vector of moving forward was higher than those in other directions. Figure 3e shows that the CMV of group T was significantly lower than that of group C (*P* < 0.05). There was no significant difference in cell movement acceleration (CMA) between the two groups (*P* > 0.05); however, the CMV and CMA of group C fluctuated widely. Figure 3f implies that *Sa* and *Ssk* were the limiting factors for the high CMV (*β* < 0, *P* < 0.05), and *Ssk* (*β* =  *− *0.51) played a more important negative role than *Sa* (*β* =  *− *0.32). However, the *Sv*, contact angle, and zeta potential did not affect the CMV (*P* > 0.05). The hydrogen content was a positive and indirect factor for high CMV (*P* < 0.05).

#### Surface micromorphological changes affected CMD

As is shown in Table 1, the morphology transformation of the titanium surface influenced the CMD. The possibility of cells moving away from the group T titanium sheet was only 13% of the probability of cells moving horizontally with the sheet compared with group C. This difference was significant (Table 1: *OR* = 0.13, *P* = 0.03 < 0.05)*.* This means that the cells on the group T titanium sheet tended to move horizontally with the sheet rather than away from it. There were no significant differences in the comparison of *Towards versus Away* and *Towards versus Horizontal.*

**Table 1:** The influence of two types of titanium sheet micromorphologies (T and C) on CMD (n = 25).

Groups	Cell movement direction (CMD)					
	Horizontal versus away		Toward versus away		Toward versus horizontal	
	*OR*	*Sig.*	*OR*	*Sig.*	*OR*	*Sig.*
T	***0.13***	***0.03***	0.63	0.46	0.40	0.16
C	1		1		1	

Significant values are in [bolditalics and underline].

The statistical significance was set at *P* < 0.05.

Within 2 $\upmu$m of the material surface, the distance between the cell and the material influenced CMD (Table 2, *P* = 0.05). Beyond 20 $\upmu$m from the material’s surface, the distance exerted no impact on CMD (Table 2, *P* > 0.05).

**Table 2:** The Influence of the vertical distance between MG-63 cells and the titanium surface on cell movement velocity (CMV) and cell movement direction (CMD) (n = 15).

Distance [$\upmu {\mathrm{m}}$]	CMV		CMD	
	*B*	*Sig.*	*B*	*Sig.*
0–20	0.82	0.51	1.16	***0.05***
21–100	0.19	0.93	2.09	0.23
101–200	1.04	0.41	2.16	0.30
> 200	0^a^		0^a^	

Significant values are in [bolditalics and underline].

Statistical significance was set at *P* < 0.05.

^a^This coefficient is set to zero because it is redundant.

### Changes in surface micromorphology affect CA

**Figure 4:**
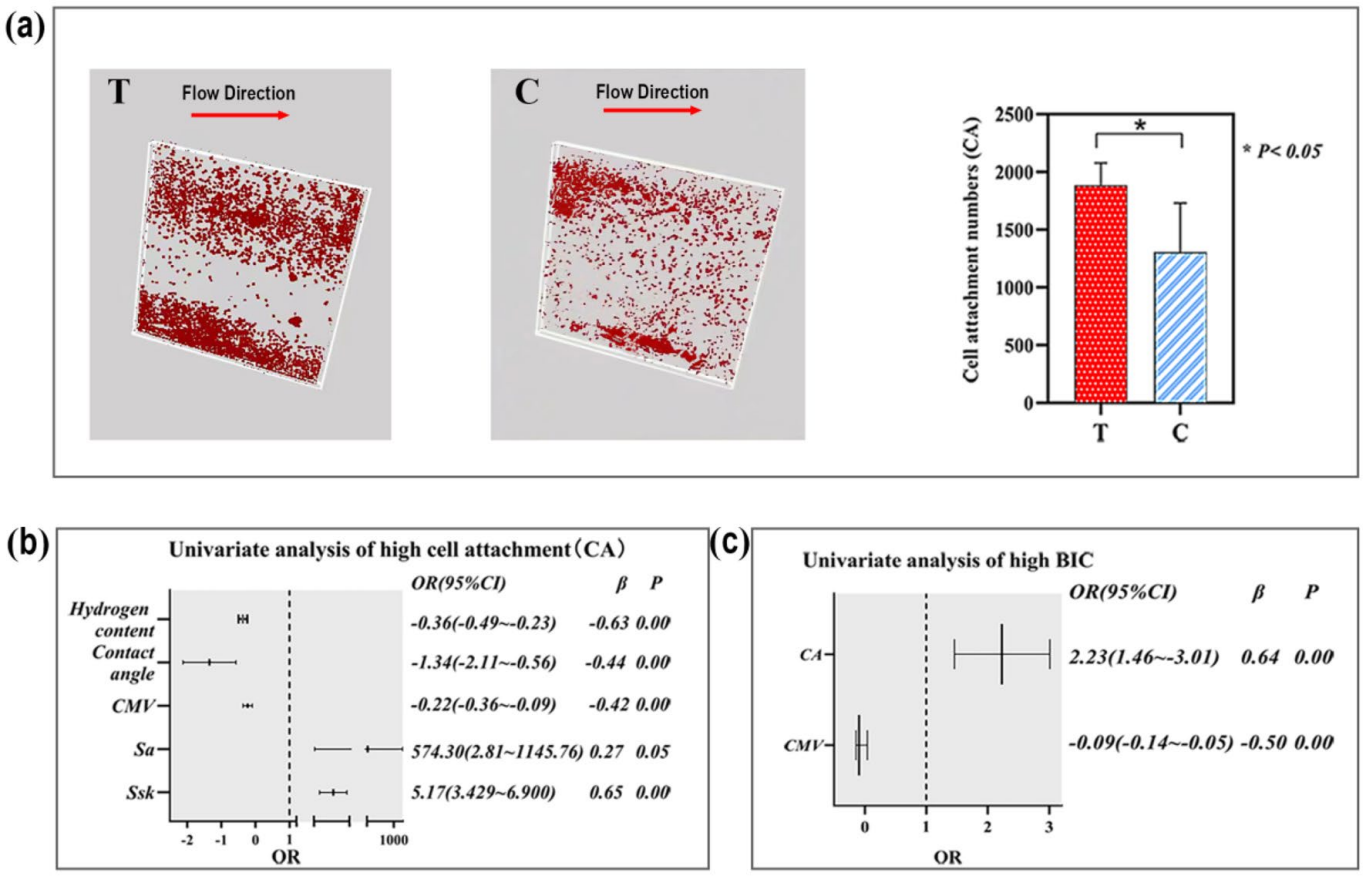
The cell movement velocity (CMV) down-regulated the cell attachment number (CA) of MG-63 cells. (**a**) Images and diagram of attached MG-63 cells at the bottom of TOC-T and TOC-C with a magnification of 20×. (**b**) Factors contributing to high CA: *Sa* and *Ssk* played positive roles in the high CA, accounting for 27% and 65%, respectively (*P* < 0.05). The CMV, contact angle, and hydrogen content were negative factors for high CA, accounting for − 42%, − 44%, and − 63%, respectively (*P* < 0.05). (**c**) The CA was a positive factor for high BIC, accounting for 64% (*β* = 0.64, *P* = 0.00); the CMV was a negative factor for the high BIC, accounting for − 50% (*β* =  *− *0.50, *P* = 0.00). The statistical significance was set at *P* < 0.05. This figure was generated by Adobe photoshop CC 2015.

As shown in Fig. 4a, the CA of group T was 1.44 times higher than that of group C (*OR* = 1.440, *P* = 0.02). Figure 4b shows that the hydrogen content, contact angle, and CMV had negative effects on cell adsorption capacity (*OR* < 1, *β* < 0.05), while *Ssk* (*OR* > 1, *P* < 0.05) and *Sa* (*OR* > 1, *P* < 0.05) had positive effects, accounting for 65.0% (*β* = 0.65) and 27.0% (*β* = 0.27) of the total weight, respectively. As indicated in Fig. 4c, CA was a positive factor for high BIC, accounting for 64% (*β* = 0.64), while CMV was a negative factor, counting for 50% (*β* = *−* 0.50) of the total weight.

## Discussion

In recent years, research on cell migration has mainly focused on activating actin, myosin-II, focal adhesion points, integrin-β1, proteolytic enzymes, and the movement of lamellar pseudopodia^25^. “Durotaxis” in stiff substrates describes how changes in tissue rigidity could play a significant role in controlling cell locomotion^26^. Hui et al. found that the cell migration speed increased when the moving channel height was reduced in a 3D microenvironment^27^. Um et al. discovered that the number of attached cells on a titanium surface with laser engraving was lower than on an untreated titanium surface. When the width of the laser engraving on the titanium surface increased from 76 to 260 μm, cell migration speeds showed no significant differences^28^. Tatkiewicz et al. found that when the slope increased gradually to 90°, the displacement of inoculated NIH-3T3 fibroblasts decreased^29^. These findings are consistent with the results of our study. In addition, Marco Salerno et al. revealed that MG-63 variability was associated with the intermediate surface roughness and feature spacing^30^. The primary limitation of this study is the lack of BIC data after the material implantation in vivo. In this study, we specifically focused on the influence of surface micromorphology of the material on the flow state of the fluid, which was closer to the real microenvironment, analyzed the roles of specific parameters of surface micromorphology in cell movement in detail, and put forward the influence range of the surface micromorphology changes on CMV and CMD.

The MG-63 cell line is a popular cell line that maintains stable osteoblast phenotypic characteristics over several passages in cell culture and is easily cultured^31,32^. Therefore, we used MG-63 cells in this study. *Sa* is used in engineering applications, whereas the equivalent *Sq* is more often used in biology. We used *Sa* values for subsequent analysis to accurately describe the surface micromorphology. In our previous study, the surface treatment method of group T was better than that of group C in terms of the early adsorption of osteoblasts^21^. As shown in Fig. 1, when the micromorphology of the material surface is altered, osteogenesis and the BIC of the material surface also change. Additionally, the BIC of the group T implant surfaces was significantly higher than that in group C. Within 2 weeks after implantation, there was no difference in BIC between the two groups. In the fourth week after implantation, the BIC of group T accelerated and was higher than that of group C. The analysis of the parameters of implant surface morphology revealed that *Ssk* and *Sa* had an impact on BIC. The probability of high BIC in implants with high *Ssk* was 2.6 times greater than that in implants with low *Ssk* (*OR* = 2.60). The probability of high BIC in implants with high *Sa* was 3.46 times higher than in implants with low *Sa* (*OR* = 3.46). With a higher *Sa*, MG-63 cells were more likely to be detained by the high steep wall, which would reduce the flow rate of the fluid, just as in group T. Thus, *Ssk* and *Sa* synergistically impacted CMV negatively, and the BIC of the implant was positively correlated to *Ssk* and *Sa* in its surface morphology. However, the hydrogen content directly affected the zeta potential on the material surface. Still, the zeta potential did not affect CMV (*OR* = 1.03, *P* = 0.68) and did not directly affect surface morphology. Therefore, traditional strong acid treatment leads to a large amount of hydrogen permeation of the material, and the amount of hydrogen permeation fails to affect the surface morphology of the material. The surface topographies depend to a great extent on the construction of the oxide film, which is oxidized by the strong acid H_2_SO_4_^22^.

Therefore, we considered whether the micromorphology of different implant surfaces would affect IOP, LBPI, and MVD and ultimately affect BIC (Fig. 2). However, the real-time detection of the IOP, LBPI, and MVD of the bone tissue adjacent to the implants showed that these did not differ among the implant surfaces. The change in surface micromorphology did not affect the IOP, LBPI, or MVD and had no significant correlation with BIC. Therefore, we speculated that the micromorphology of the implant material only affects adjacent cells. We used canine jaws with acellular treatments and implanted titanium sheets with the same treatment as the implant surface into the microchannel. MG-63 cells were prepared as single-cell suspensions, which were pumped into the chip by the velocity of posterior alveolar artery blood flow using the microflow control system to study the motion characteristics of single MG-63 cells (Fig. 3a,b). The analysis of the movement speed and trace of the cells on the surface of the two groups of materials showed that the CMV in group C was higher than that in group T (Fig. 3d). The cell movement on the surface of group T tended to move horizontally rather than away from the material surface (Table 1). However, the change in the cell movement direction was caused by the change in surface morphology only when the cell was close to the material surface (within the < 20 μm range) (Table 2). This result agrees with the results of recent studies^28^.

**Figure 5:**
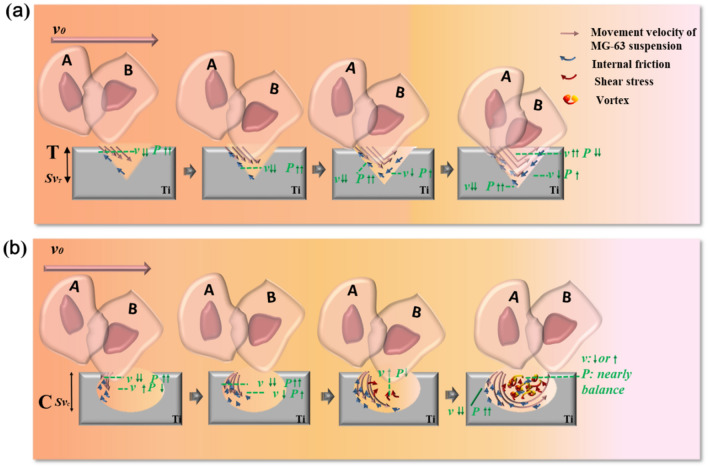
Schematic diagram of the processes of MG-63 cells attached to the surfaces of group T (**a**) and group C (**b**). The MG-63 cells were more likely to be adsorbed onto the material surface of group T than group C. *v*: cell velocity; *P*: air pressure. This figure was generated by Adobe photoshop CC 2015.

The MG-63 suspension can be regarded as a Newtonian fluid owing to its homogeneous characteristics. The flow state of the suspension entering the TOC was laminar owing to the low velocity (9.0 μL min^−1^). The fluid we studied was in the boundary layer range and was subject to sliding friction caused by the material surface and internal friction caused by the fluid’s viscosity. The longitudinal section of the groove was a deep “V” shape, as the *Ssk* of the group T surface was 0.817 ± 0.232 (0° < α_T_ < 90°). This surface trait contributed to the general stability of CMV, although this either increased or decreased slightly when the fluid entered a smaller or larger space (Fig. 5a). Therefore, cells were more likely to be adsorbed onto the material surface (Fig. 5a: cells A and B). The longitudinal section of the surface micromorphology of group C is a major arc (*Ssk*_*C*_ = − 0.297 ± 0.153, 90° < α_C_ < 180°); when the fluid flowed through the surface of group C, a small vortex was formed. During this time, the velocity at the center of the vortex increased and decreased simultaneously. The air pressure was nearly balanced, and the cells were far away from the material surface (Fig. 5b: cell A), leading to reduced cell adsorption. When the cell contacted the concave boundary, it could attach to the surface (Fig. 5b: cell B). This result agrees with that of a recent study^29^.

When the fluid or suspension enters an extremely narrow channel in which the area of the liquid is located within the boundary layer, the movement velocity of the fluid or suspension decreases significantly. However, we found that when the fluid entered the channel that was slightly narrower than the wider space, the velocity of the suspension that was distal to the bone wall and material surface increased, while the suspension velocity closest to the surface of the material remained the lowest, indicating that the titanium sheet can only affect the cells that move along the material surface. In contrast, the cells distal to the surface were influenced by the form or diameter changes of the passageway in the local bone. Therefore, the CMD alternation of the cells that lie within the area further than 20 $\upmu$m away from the material surface should be attributed to the transformation of the bone microstructure but not to the change in titanium surface morphology.

In implant surgery, some scholars have advocated bone compression in areas with low bone density (class IV bone in the maxillary posterior tooth area) to improve bone mineral density. When the large bone trabecular space becomes smaller, local blood flow speed is accelerated, which is unfavorable for the adhesion of osteoblasts to bone. Class I–II bone, consisting of much cortical and plate-shaped bone, is compressed (this technique is often used when mandibular implants need to obtain good initial stability to facilitate immediate loading), which would further squeeze the bone trabeculae into close contact. A torque of 30 *Ncm* is enough to displace or even fracture the bone trabeculae when the Tb.th is less than 0.5 mm (Fig. 2c). Owing to blood viscosity, the blood flow movement is completely localized in the boundary layer, which leads to an extremely low flow rate, eventually leading to local ischemia, bone death, and implant failure. The correct technique does not artificially change the inherent structure of bone tissue but achieves good BIC by inducing programmed reconstruction of bone trabeculae.

The main factors influencing IOP and LBPI are arterial pressure, venous reflux, blood volume, and passageway volume of the blood passing through. As none of these factors were altered by the variation in material surface morphology, morphology could not affect the balance of IOP, LBPI, and MVD.

The CA results were consistent with results obtained in one of our previous studies^23^. Therefore, at the cellular level, the surface morphology of group T was more conducive to the promotion of cell attachment.

It can be concluded that the *Ssk* and *Sa* of the implant surface micromorphology could positively affect the movement velocity and direction of MG-63 cells close to the surface (< 20 $\upmu {\mathrm{m}}$), and then positively affect BIC. As the *Ssk* and *Sa* values of the implant surface of group T were higher than those of group C, the osteogenesis ability of group T was better than that of group C. The surface morphology of the material cannot affect cell movement or hemodynamic factors far from the material surface (> 20 $\upmu {\mathrm{m}}$). The CMV and CMD alternation of the cells that lay within the area further than 20 μm away from the material surface should be attributed to the transformation of the bone microstructure.

## Materials and methods

### Titanium implant preparation

The grade IV titanium implants (Carpenter, US) had an identical cylindrical shape with a core diameter of 2.0 mm and three rings with an outer diameter of 3.5 mm and a length of 7.8 mm (Fig. 1a). The titanium sheets were reduced to 4.0 × 2.0 × 0.2 mm, polished, and acid-etched by 4.62 mol L^−1^ H_3_PO_4_ + 0.249 mol L^−1^ NaF or 5.00 mol L^−1^ H_2_SO_4_ + 5.05 mol L^−1^ HCl, respectively, at 60 °C for 30 min. After etching, the samples were cleaned with acetone and anhydrous alcohol for 30 min each. *Ssk, Sa*, surface concave depth (*Sv*), contact angle, and hydrogen content were examined using white-light interferometry (Taylor Hobson CII, Leicester, United Kingdom), a surface contact angle tester (Dataphysics OCA40, Germany), and inert gas fusion thermal conductivity analysis (ASTM E 1447-09), respectively (n = 25).

### Implant implantation

Eighteen healthy 1-year-old beagle dogs, with no infectious or circulatory system diseases and weighing 13–16 kg, and inoculated as required, were purchased from and kept in the Guangdong Medical Animal Experimental Center, with license no. syxk (Yue) 2018-0003. The dogs were kept and fed in single cages, and a 12 h:12 h day-night photoperiod was employed. The beagle dogs had ad libitum access to water and on-demand access to food. The contents and procedures related to the animal test were approved by the Animal Ethics Committee of the Guangdong Medical Animal Experimental Center (C2020-05-11). This project was performed according to the provisions of the Animal Welfare Act, PHS Animal Welfare Policy, the principles of the “NIH Guide for the Care and Use of Laboratory Animals,” and the updated ARRIVE 2.0 guidelines (the ARRIVE 2.0 guideline checklist has been adhered to and uploaded).

The experiment was conducted in a comparative medicine laboratory. The animals were prohibited from drinking and eating for 16 h prior to surgery. One day before the surgery, 200,000 units of penicillin G sodium were injected intramuscularly. The beagle dogs were weighed and anesthetized by an intramuscular injection of 0.06 mL kg^−1^ of xylazine hydrochloride (Shengxin, 20180201) and an intraperitoneal injection of 3% pentobarbital sodium solution 6 mg kg^−1^ (Sigma, 0123A001), respectively. Then, local infiltration anesthesia of their bilateral premolars with 2% lidocaine was performed. The first, second, third, and fourth premolars of the bilateral mandibles of the beagle dogs were extracted. The experiments were initiated 2 months after tooth extraction. Before the experiments, three dogs were used as the blank controls; these were anesthetized with intraperitoneal injections of a 3% sodium pentobarbital solution (6 mg/kg), euthanized by exsanguination, and then subjected to treatment.

The implants were implanted in the second, third, and fourth premolar areas of the bilateral mandible (right mandible: T1, T2, and T3; left mandible: C1, C2, and C3) using a surgical guide (SI-923; W&H, Australia) (Fig. 2a). After IOP and LBPI detection, we monitored the physiological condition of the dogs until they recovered from anesthesia before returning them to their home cages. The dogs were treated with penicillin G sodium (400,000 units kg^−1^) for 3 days after surgery. After implantation, three dogs were euthanized at each time point (3 d, 1 w, 2 w, 4 w, 6 w, n = 3).

### IOP detection

The three-way valve of the biological function system (BL-420S; Chengdu Taimeng, China) was connected to a pressure sensor, syringe, and bone puncture needle. The bone puncture needle was inserted at the root tip of the fourth premolar (Supplementary file 3a). The height of the pressure sensor was adjusted to the same height as the heart position of the beagle dog, and it was maintained for 10 min after the pressure stabilized (Supplementary file 3b). The index was measured and recorded before implantation and 3 d, 1 w, 2 w, 4 w, and 6 w after implantation. To avoid errors, each post-implantation detection site was consistent with the preimplantation detection site.

### Local blood perfusion index (LBPI)

LBPI was measured using a laser Doppler blood flow detection system (Perimed, Sweden) (Supplementary file 3c). The optical fiber probe was maintained for 3 min after the value was stable. The time points and sites of the index measurements were the same as those for IOP.

### Analysis of bone morphology and densitometry

At 3 d, 1 w, 2 w, 4 w, and 6 w after implantation and before implantation, 15 dogs were euthanized, and a harmless treatment was carried out at five time points. The implants and surrounding bone were examined by microscopic CT (Skyscan1172, Bruker, Germany). BMD, BV/TV, SMI, DA, Tb. N, Tb.Th, and Tb. Sp was analyzed using CTAn^33^. Mimics (Belgium) was used for image processing (n = 3).

### Pathological observation and MVD of newly bone formed

The fixed samples were dehydrated, vacuumized, embedded, sliced using a hard tissue slicer (Leica SP1600, Germany), polished, washed, stained with methylene blue acid fuchsin, and observed under an optical microscope (at magnifications of 20× and 40×). The BIC of each implant was calculated (n = 3).

The samples were decalcified by EDTA for 2 months, dewaxed, hydrated, and antigen repaired was conducted by treatment with hyaluronidase at 37 °C for 30 min; washed three times with phosphate buffered saline for 3 min; pepsin treatment at 37 °C for 30 min; and finally washed with phosphate buffered saline for 3 min, three times. Then, freshly prepared 3% hydrogen peroxide was added to remove the endogenous peroxidase blocking solution and incubated at 25 °C for 10 min. A 5% bovine serum albumin solution was dropped on the slide and incubated at 37 °C for 30 min for blocking. The samples were subsequently incubated with the primary antibody CD34 (1:200) (ab81289, Abcam, UK) overnight at 4 °C and incubated with horseradish enzyme-labeled goat anti rabbit IgG (H + L) (1:200) (ZB-2301, ZSGB) at 37 °C for 30 min and developed with DAB for 5–10 min (cw0125, Cwbio, China). They were then redyed using hematoxylin for 3 min (ar1180-1, Biolead, China), and were observed using a forward bright-field microscope (Leica DM2500, Germany) at a magnification of 200×.

### Detection of characteristic MG-63 movement in the TOC

#### Preparation of ACAB and the fabrication of the TOC

Canine cancellous bone was prepared as a 3.0 × 4.0 × 4.0 mm bone mass and washed with deionized water. The bone was kept in 10% NaCl and 0.5% Triton X-100 (Sigma) at a volume ratio of 1:1 at room temperature for 1 w. The TOC passage diameter was 500 $\upmu {\mathrm{m}}$^34^. Grade four titanium sheets (Carpenter, US) were reduced to 4.0 × 2.0 × 0.2 mm, then polished and acid-etched using the two different acid etching formulas. The *Ssk, Sa, Sv,* contact angle, and hydrogen content were measured as 4.1. Polydimethylsiloxane (PDMS) was used to fix the titanium sheet and bone block according to the design, and the preparation was sterilized (Fig. 3a).

#### Instrument connection and installation

The entire pipeline connection included a tissue-on-chip (Young Chip, China), an air compressor (550w-8L, OTS, China), a mechanical air valve (S3Y-06, Air TAC, Taiwan, China), and a peristaltic pump (KSP-F01A, Kamoer, China). The ELVEFLOW microfluidic pressure and flow control system included a pressure controller (Fluidic Lab), a flow sensor (Fluidic Lab), and the software ELVEFLOW Smart Interface (ESI) chose the pressure channel: OB1 MK3 + (ELVEFLOW, France). The connection mode is illustrated in Fig. 3b.

#### Fluorescent labeling of the MG-63 cell membrane

MG-63 cells (Procell, CL-0157, China) were cultured with 10% fetal bovine serum (Gibco, 16000044) in DMEM (Gibco, US). When the fusion rate of MG-63 reached 80–90% at the third passage, the cells were digested with 0.25% trypsin + EDTA (Gibco, 25200056), resuspended, and adjusted to 5.0 × 10^4^ mL^−1^, and centrifuged for 5 min at 400 × *g* min^−1^. Under dark conditions, 1 mL of diluent C was added to the MG-63 residue, mixed with a 2 μL PKH26 cell linker fluorescent staining solution (PKH26, MIDI26, Sigma, Germany) and 1 mL of diluent C, and incubated for 5 min. Next, 2 mL of DMEM low glucose medium containing 10% FBS was added to stop the reaction. After 2 mL of D. Hanks solution was added and centrifuged twice, the MG-63 suspension with a concentration of 5.0 × 10^4^ mL^−1^ was prepared. The excitation wavelength was 551–567 nm.

#### Detection of MG-63 movement in the TOC

An MG-63 suspension (2 mL) stained with PKH26 was loaded into a liquid storage tank and injected a 9.0 μL min^−1^^20^. The 90 min moving process of MG-63 in the TOC-T and TOC-C was observed and recorded using a particle tracking velocimetry system (PTVS). The CMV and CMA were analyzed at different time points using Dynamics Studio 6.8 (Dantec Dynamics, Demark).

#### Laser confocal observation of cell adsorption

After the experiment, the TOC was injected with neutral formaldehyde at 9.0 μL min^−1^, fixed for 15 min, and observed using a laser confocal microscope (TCS SP8 X, Leica, Germany) for the number of cells adsorbed at a magnification of 20×.

#### Flow field analysis

ACAB and titanium sheets from the two groups were scanned using microscopic CT (Skyscan1172, Bruker, Germany) for 3D reconstruction. A viscometer determined the viscosity of the MG-63 suspension to be 195.909 ± 14.273 mpa s^−1^ (Brookfield, NDJ-5S, US). The fluid speed at the entrance was 9.0 μL min^−1^. The fluid velocity of the TOC flow field was analyzed using Workbench 19.0 and Fluent 19.0.

### Statistical methods

Data were analyzed using SPSS 25.0. Continuous numerical variables were analyzed using repeated-measures analysis of variance, simple effect analysis, and linear regression. Ordered categorical variables were analyzed using the generalized estimation equation (GEE) with a detection level of α = 0.05. The statistical significance was set at *P* < 0.05. The results are also reported as odds ratios and 95% confidence intervals: *OR* (95% *CI*).

### Ethics approval

The procedures related to animal testing were approved by the Animal Ethics Committee of Guangdong Medical Animal Experimental Center (C2020-05-11) and carried out according to the updated ARRIVE 2.0 guidelines.

## Supplementary Information

Supplementary Video 1.

Supplementary Video 2.

Supplementary Video 3.

Supplementary Video 4.

Supplementary Information 1.

Supplementary Information 2.

Supplementary Information 3.

## Data Availability

All data generated or analysed during this study are included in this published article [and its supplementary information files 5].
