# Supplementary figures and images for: The role of titanium surface micromorphology in MG-63 cell motility during osteogenesis
Source: Sci Rep. 2022 Jun 15;12:9971. doi: 10.1038/s41598-022-13854-2 (PMC9200830; doi:10.1038/s41598-022-13854-2)

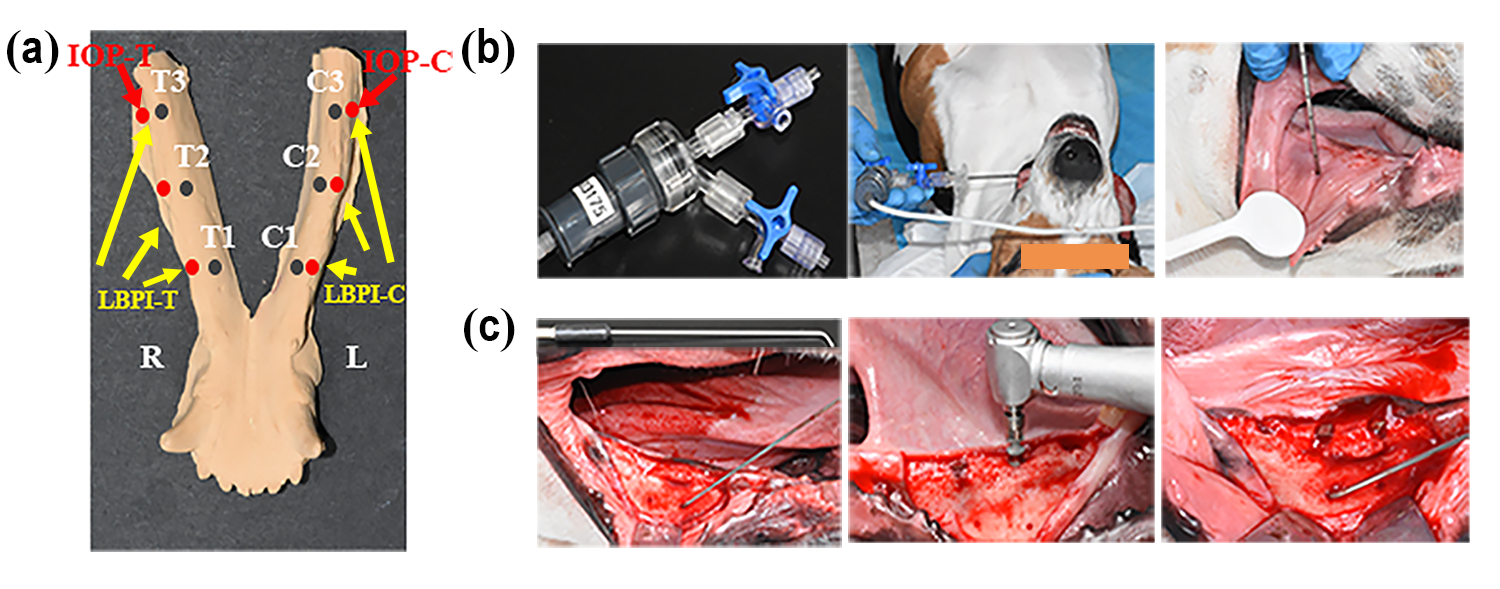

Supplement: Supplementary file 5 — Supplementary Information 1. [file 41598_2022_13854_MOESM5_ESM.tif]

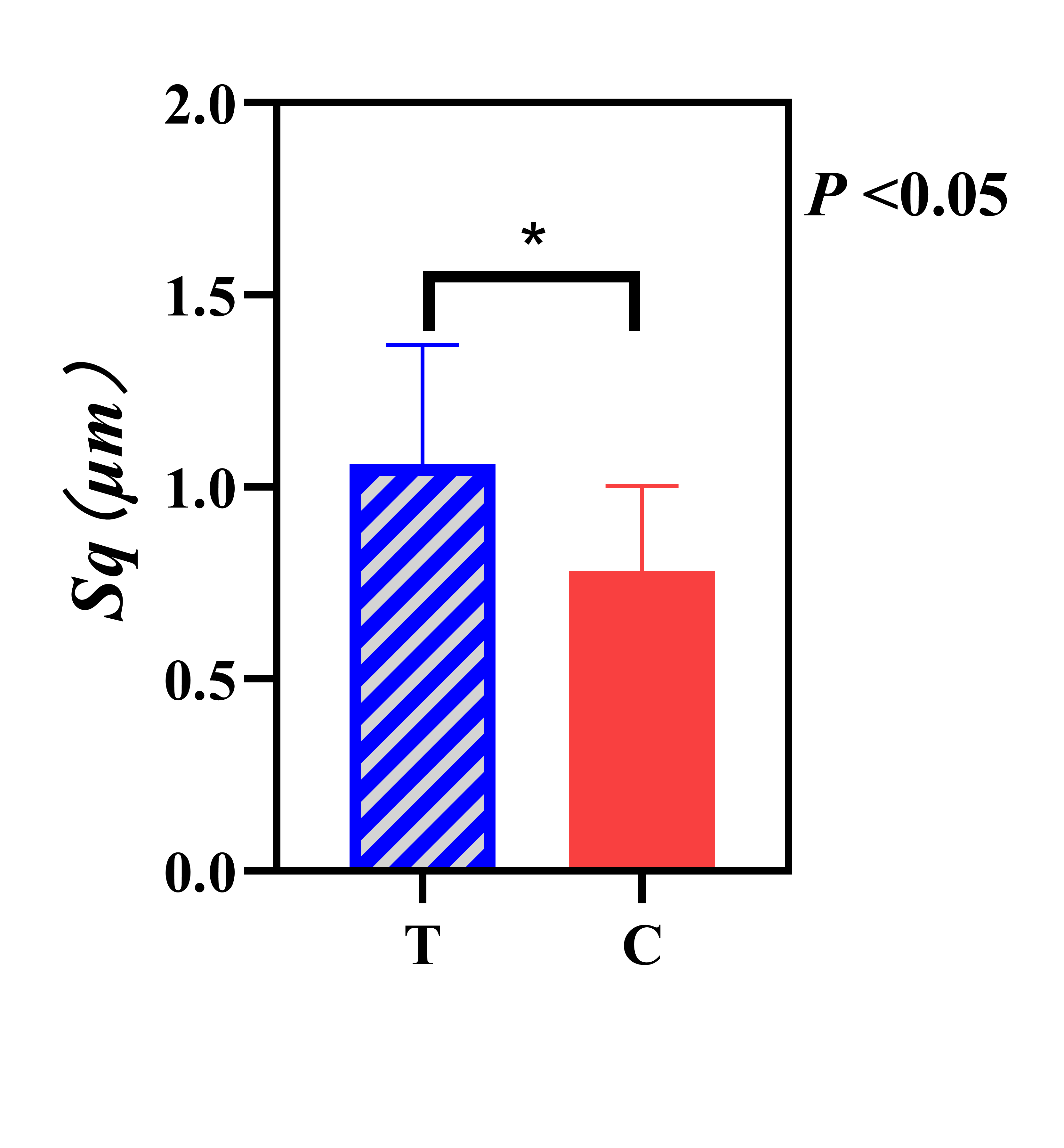

Supplement: Supplementary file 6 — Supplementary Information 2. [file 41598_2022_13854_MOESM6_ESM.tif]
